# Recent Uses of Paper Microfluidics in Isothermal Nucleic Acid Amplification Tests

**DOI:** 10.3390/bios13090885

**Published:** 2023-09-15

**Authors:** Jocelyn Reynolds, Reid S. Loeffler, Preston J. Leigh, Hannah A. Lopez, Jeong-Yeol Yoon

**Affiliations:** 1 Department of Biomedical Engineering, The University of Arizona, Tucson, AZ 85721, USA; jocelynreynolds@arizona.edu (J.R.); reidloeffler@arizona.edu (R.S.L.); pleigh@arizona.edu (P.J.L.); 2 Department of Neuroscience, The University of Arizona, Tucson, AZ 85721, USA; mhanlopez@arizona.edu

**Keywords:** recombinase polymerase amplification, loop-mediated isothermal amplification, rolling circle amplification, lateral flow immunochromatographic assay, microfluidic paper-based analytic device

## Abstract

Isothermal nucleic acid amplification tests have recently gained popularity over polymerase chain reaction (PCR), as they only require a constant temperature and significantly simplify nucleic acid amplification. Recently, numerous attempts have been made to incorporate paper microfluidics into these isothermal amplification tests. Paper microfluidics (including lateral flow strips) have been used to extract nucleic acids, amplify the target gene, and detect amplified products, all toward automating the process. We investigated the literature from 2020 to the present, i.e., since the onset of the COVID-19 pandemic, during which a significant surge in isothermal amplification tests has been observed. Paper microfluidic detection has been used extensively for recombinase polymerase amplification (RPA) and its related methods, along with loop-mediated isothermal amplification (LAMP) and rolling circle amplification (RCA). Detection was conducted primarily with colorimetric and fluorometric methods, although a few publications demonstrated flow distance- and surface-enhanced Raman spectroscopic (SERS)-based detection. A good number of publications could be found that demonstrated both amplification and detection on paper microfluidic platforms. A small number of publications could be found that showed extraction or all three procedures (i.e., fully integrated systems) on paper microfluidic platforms, necessitating the need for future work.

## 1. Introduction

In recent years, extensive endeavors have been made to create devices capable of identifying DNA or RNA sequences from a wide variety of samples types. Since DNA and RNA are the essential building blocks that encode all forms of genetic information, nucleic acids are important biomarkers that can be used to detect a myriad of targets accurately and quickly. Since the nucleic acids in any given sample are extremely low in number, they should be duplicated through multiple iterations, known as the nucleic acid amplification test (NAAT). Such NAAT detection can be utilized for agricultural reproduction [1], food safety [2], and clinical diagnostics [3], to name a few. One of the most popular NAATs is polymerase chain reaction (PCR), consisting of three key steps: denaturation, annealing, and extension. Each step occurs at different temperatures and requires a thermal cycler to shift between them. The double-stranded (ds) DNA splits into two single-stranded (ss) DNAs upon denaturation (typically at 94–98 °C); the primers attach to the complementary sequences during annealing (typically at 55–72 °C); and finally, the DNA polymerase synthesizes the double-stranded DNA starting from the primers during the extension (typically at 72 °C) [4,5]. These three steps comprise one cycle, generating two identical copies from an original target. Repeating it for 30 cycles can theoretically produce 2^30^ copies or approximately 10^9^ copies.

PCR is the first NAAT method and remains the most popular method for amplifying and detecting nucleic acids. PCR has recently gained sweeping popularity among the general public due to its widespread use for COVID-19 detection during the pandemic [6,7]. Although it may be the first and most popular method, several drawbacks have been identified. For example, PCR has lower specificity than other NAATs, can only use a narrow range of specific primers, and is less cost-effective due to the specialized equipment and required training [8,9,10]. One of the most significant inhibiting factors of PCR is the heavy dependence on temperature control equipment, i.e., a thermal cycler, which is the reason for developing isothermal methods.

Isothermal nucleic acid amplification tests (often abbreviated as isothermal amplification tests) have been developed as an alternative to PCR. Since the reactions are conducted at a constant temperature (=isothermal), they do not require the complex thermal cycling necessary for PCR [11]. This makes them more accessible, cost-effective, and faster, greatly favoring point-of-care (POC) testing [12]. Moreover, the speed of isothermal amplification tests is determined primarily by the enzyme activity, while that of PCR depends on the temperature ramp rate (°C/s; how fast the temperature can be changed from one step to another). Consequently, attempts to demonstrate isothermal amplification tests in microfluidic devices are unlikely to shorten the amplification time, as achieved in microfluidic PCR through heat transport optimization. In response, isothermal amplification tests are being developed to miniaturize the systems, e.g., in microchambers, without fluidic motion during reactions. This simple design grants isothermal amplification tests a lower energy requirement (no need for rapid heating, cooling, or re-circulation), making it particularly appealing for battery-operated portable systems [13].

The recent development of paper-based diagnostic technologies, such as lateral flow immunochromatographic assays (LFIA; commonly known as rapid tests) and paper-based microfluidic devices, have evolved to be applied to isothermal amplification tests aimed toward field-based pathogen detection and POC diagnostics. A paper consists of overlain cellulose fibers that create pores where fluid can flow through capillary action due to the adhesive forces between the liquid and the surfaces [14]. The spontaneous flow (without needing an external pump or a separate power supply) makes it desirable for microfluidic applications. Many techniques are available for designing and fabricating paper-based microfluidic devices [15], where all consist of creating some hydrophobic boundary region in which the fluid flows within the pre-defined channel in one direction.

There are multiple benefits to using paper in microfluidics, including (1) no need for external forces to drive fluid flow as mentioned previously; (2) extensive accessibility and low cost of paper; (3) fast, inexpensive, and simple manufacturing of paper microfluidic chips, requiring minimal to no laboratory tools or specialized environments [16]; (4) potential for long-term storage, including those containing biological materials [17]; (5) compatibility with many chemicals and organic materials; and (6) ease of disposal through biodegradability or incineration [18]. Unique patterns of channels can be printed on paper to control time-dependent reactions, delay/separate/shut off the fluid flow, and allow for multiplex detection [19,20,21], making the paper a versatile tool. These properties make paper microfluidic devices advantageous as analytical tools, known as microfluidic paper-based analytic devices (μPADs), for field testing and clinical POC use, and indeed for NAATs. Research shows that paper-based diagnostics can meet the World Health Organization’s ASSURED criteria for ideal POC devices, i.e., affordable, sensitive, specific, user-friendly, rapid and robust, equipment-free, and deliverable to end-users [22]. ASSURED criteria are critical in resource-deficient settings where accesses to laboratory equipment, trained professionals, and specialized environments are limited. Many applications of paper-based NAATs have been demonstrated in field and POC use, such as pathogen detection in food or water [23], human immunodeficiency virus (HIV) viral load testing [24], and, more recently, SARS-CoV-2 testing [25].

An earlier review of paper microfluidics for NAATs discussed the progress and limitations as of 2017 [26]. It explained that sample preparation was the most extensive, requiring cell lysis, nucleic acid extraction, and sample purification to remove any reaction inhibitors. Furthermore, sample preparation involved many steps and reagents, so it has often been performed off the paper platforms. Unfortunately, this makes these devices less desirable for fieldwork. However, some recent publications in this review demonstrated paper-based sample extraction, while others have developed all-in-one devices capable of extraction, amplification, and detection. Furthermore, Kaur et al. [27] reported that the grand challenges of paper-based microfluidics were the compromised amplification efficiency, dry storage of isothermal nucleic acid amplification reagents altering the melting temperature of the DNA and thus impacting the reaction efficiently, and along similar lines, rehydration of these dried reagents. When the immobilized reagents are rehydrated on paper, the reaction cannot be thoroughly mixed, creating regions of high and low concentration. The uneven distribution of the sample can then alter the detection, especially in LFIAs. While both of these earlier reviews provide insight into some of the current challenges with paper microfluidic NAATs, neither compares nor summarizes what has been carried out across various amplification methods, which is the aim of this review.

Since the global COVID-19 pandemic caused by SARS-CoV-2, paper microfluidic-based antigen tests and NAATs have been widely developed toward at-home, self-testing, and POC diagnostic methods, due to the fast production time, automated procedure, and the ease-of-use of paper platforms. NAATs were one of the primary testing methods for different strains of SARS-CoV-2 and continue to remain relevant as more variants arise even today. As addressed previously, isothermal amplification tests are well suited to paper microfluidic platforms, as they do not require the rapid and precise temperature modulation seen in PCR. The ease of use and simplicity of paper-based isothermal amplification tests have opened the door for innovation and future potential for commercialization.

Paper microfluidics have been used toward isothermal amplification tests for three purposes. (1) Extraction: the porosity of paper makes it well suited to separate particles from each other, which can extract and purify nucleic acids from biological samples [28]. (2) Amplification: isothermal nucleic acid amplification can also be performed on paper microfluidic devices by creating a ready-to-use device with pre-immobilized reagents within the paper fibers [29]. (3) Detection: bioreceptors, such as gold nanoparticles, enzymes, intercalating dyes, and oligonucleotide receptors, can also be pre-immobilized within the paper fibers to detect, qualify, and quantify the amplification progress or the presence of target molecules. Numerous detection methods have been demonstrated for isothermal nucleic acid amplification tests, such as colorimetric, fluorometric, flow distance, spectroscopic, etc. [30]. An earlier review looked at the current developments in paper-based NAATs as of 2018 while categorizing the devices into three categories: direct amplification, semi-integrated, and fully integrated amplification [27]. This review noted that when searching paper-based nucleic acid amplification, many articles performed nucleic acid extraction or detection via LFIA strips. At the time of publication, amplification performed on paper was limited. In recent years, developments in paper-based amplification have increased, whereas limited progress has been made in paper-based extraction. As seen in our review paper, the detection on paper was the most widely used aspect of nucleic acid testing.

While many isothermal amplification tests have been developed, the following methods have been demonstrated in association with paper microfluidic devices: RPA (recombinase polymerase amplification), RAA (recombinase-aided amplification), MIRA (multienzyme rapid amplification), LAMP (loop-mediated isothermal amplification), RCA (rolling circle amplification), NASBA (nucleic acid sequence-based amplification), HDA (helicase-dependent amplification), SDA (strand displacement amplification), EXPAR (exponential amplification reaction), and CAMP (competitive annealing mediated isothermal amplification). We searched Google Scholar for the full names of these isothermal tests with paper-based microfluidics or lateral flow and filtered the search results from 1 January 2020 to 1 July 2023, i.e., 3.5 years, representing the pandemic and post-pandemic (endemic) periods. Identified journal articles were manually inspected to check whether the paper microfluidic chips were used with the isothermal amplification tests as their primary research objectives. Furthermore, no review papers were selected as key references when manually inspecting potential articles.

**Figure 1:**
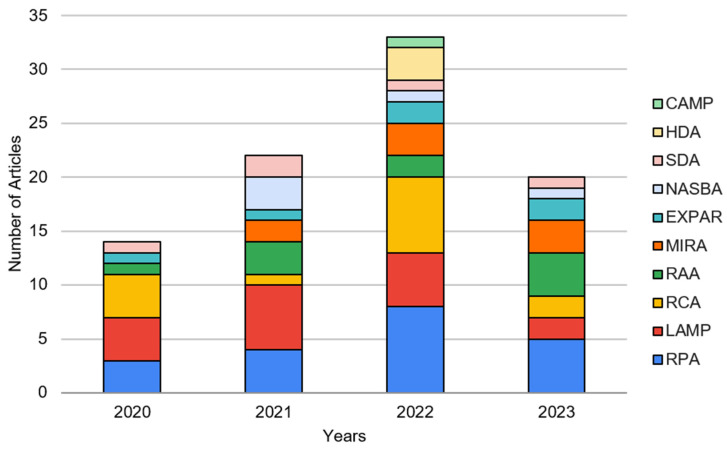
The number of journal articles demonstrating paper microfluidics (including lateral flow assay) for various isothermal amplification tests, sorted by year, from 1 January 2020 to 1 July 2023.

Figure 1 summarizes the results, showing that RPA and LAMP are the two most studied isothermal tests associated with paper microfluidics or LFIA. RAA and MIRA, which are related to RPA, have also gained in popularity. RCA has also received attention in recent years. Therefore, we categorized the isothermal amplification tests into (1) RPA, (2) RAA/MIRA, (3) LAMP, (4) RCA, and (5) others in the following sections.

The general trends of these articles are supported by previous review articles. For example, in [27], a similar yearly publication graphic was created to represent the number of papers between 2009 and 2017. The authors determined that during that time interval, LAMP was the most common amplification method and, in 2016, had the greatest number of publications, 12 articles. Another review paper [26] analyzed 15 articles published between 2006 and 2017 on paper microfluidic nucleic acid amplification, and 6 of the 15 articles performed LAMP, while three utilized RPA. The findings from previous reviews are consistent with the analysis presented in this review. LAMP is still a predominant application method investigated with paper microfluidics; however, there is a growing interest in RPA. Another recently published review on paper-based LAMP [31] generated a similar figure. It shows a slow increase in publications from 2015 to 2020; however, from 2020 to 2021, there is a significant increase in the number of publications, affirming that the SARS-CoV-2 pandemic initiated more development in this research field.

**Figure 2:**
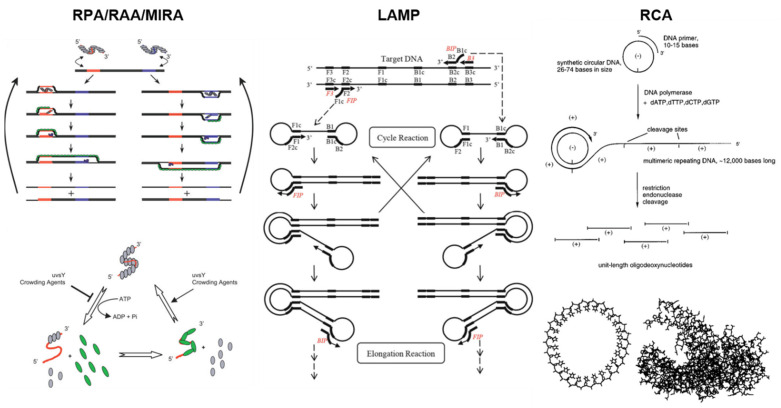
Working principles of RPA/RAA/MIRA (**left**) describe the scanning of template DNA for homologous sequences via RPA recombinase and primers. Once identified, the strands are bound to gp32 (green), while the primers are lengthened via the Bsu polymerase (blue), generating one complete copy of the original template. As the process continues, this will result in increased DNA amplification. To develop a favorable environment, in the presence of ATP, the recombinase uvsX (gray) binds to oligonucleotides (red). However, during the process of ATP hydrolysis, the disassembly of the uvsX–oligonucleotide complex will initiate the binding of oligonucleotides with gp32 (green), which is a DNA-binding protein crucial to the recombinase–primer reaction. The working principle of LAMP (**middle**) is composed of forward inner primers (FIP) and backward inner primers (BIP) that self-elongate in a loop structure. These elongations are repeated with DNA polymerase. Strand displacement synthesis increases the amounts of DNA amplification products and complementary sequences. The working principle of RCA (**right**) consists of circular oligodeoxynucleotides that are templates for the DNA polymerases. They can be thousands of bases long; however, these may also be cleaved into monomer-sized oligonucleotides. For size comparison, a circular oligodeoxynucleotide 26-bases in length and a structure of DNA polymerase are depicted, specifically the Klenow fragment of *E. coli*. Reprinted from [32] under Creative Commons Attribution License. Reprinted with permissions from [33], Copyright 2015 Springer Nature, and [34], Copyright 1996 American Chemical Society.

The working principles of RPA/RAA/MIRA, LAMP, and RCA are depicted in Figure 2.

These journal articles demonstrated the use of the paper microfluidics (including LFIA) for extraction, amplification, detection, amplification + detection, and all three (fully integrated systems). Most reports demonstrated detection, and were further categorized based on their detection methods: colorimetric, fluorometric, flow, surface-enhanced Raman spectroscopic (SERS), and others. The following sections discuss paper-based extraction, amplification, and detection for each isothermal amplification category.

## 2. RPA with Paper Microfluidics

Recombinase polymerase amplification (RPA) is an isothermal method that utilizes recombinase enzyme. This enables successful strand displacement (without heating up to the denaturation temperature of 94–98 °C) and subsequent insertion of primers at cognate sites. Primer ejection via branch migration is prevented by ssDNA binding proteins that stabilize recombinase–primer complexes. After the recombinase is disassembled, the 3′-end of the newly inserted primer is available for strand-displacing DNA polymerase binding. Primer extension is followed, and exponential amplification is achieved by repeating these steps [32]. The RCA process is depicted in Figure 2 on the left. While the other isothermal amplification methods require many primers (LAMP typically requires six primers), RPA requires only two primers [32,33]. Additionally, RPA can be conducted at 37 °C, avoiding the high temperatures needed by other isothermal amplification platforms [32]. Another benefit of RPA is its ability to amplify products to detectable levels in as little as 10 min while maintaining a reaction temperature of 37 °C [35]. Figure 1 shows that RPA has the highest number of associated articles for 2020–2023, which is not surprising given the numerous advantages of this method. The combination of low reaction temperature with rapid amplification makes RPA a great candidate for portable and point-of-care systems.

**Figure 3:**
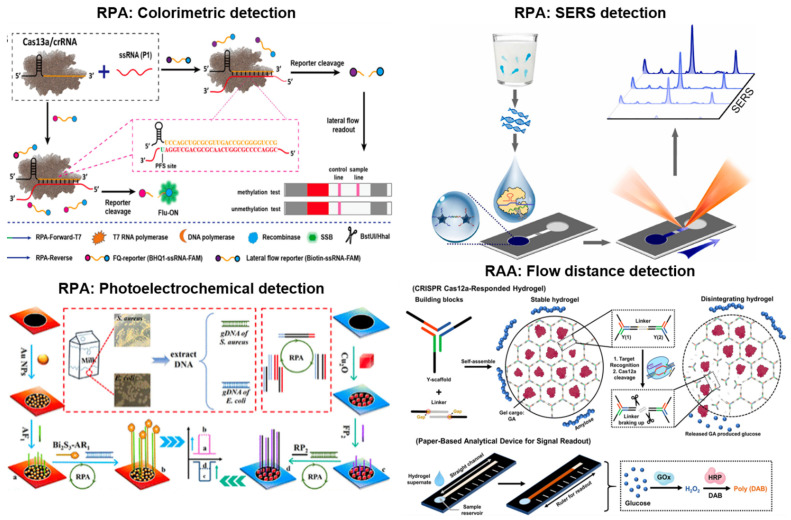
Paper-based detection examples with RPA and RAA. Colorimetric detection (**top left**) is demonstrated using RPA and CRISPR/Cas13a to determine DNA methylation in a site-specific manner. It shows the signal amplification for methylated DNA and the elimination of the false identification of incomplete breakdown of unmethylated targets. SERS detection (**top right**) is demonstrated for RPA-Cas12a on a microfluidic paper-based analytical device (μPAD), increasing sensitivity in a portable, qualitative manner. Photoelectrochemical detection (**bottom left**) is shown on 3D-printed, paper-based electrodes to rapidly detect foodborne pathogens’ nucleic acids under 980 nm light. In the flow distance detection (**bottom right**), with crRNA guidance, target dsDNA becomes actuated and cleaves linkers within the DNA hydrogel. Once the linkers are broken down, signal molecules are released in the hydrogel to achieve signal transduction. As denoted in the figure, glucose is oxidized into hydrogen peroxide within the circular reservoir and converted into poly(DAB) in the straight channel via HRP. Visibly, the distance of the bar indicates the content of rRNA. Reprinted with permissions from [48], Copyright 2021 American Chemical Society; [49], Copyright 2022 Elsevier; [52], Copyright 2023 American Chemical Society; and [67], Copyright 2021 American Chemical Society. Note: [52] demonstrated amplification in addition to detection.

Most RPA systems utilize lateral flow immunochromatographic assay (LFIA) strips. Because of this, colorimetric is the most common detection method among RPA assays. For LFIA detection, probes and/or primers are tagged, generating tagged amplicons. These tagged amplicons are loaded onto the strip with a bioreceptor (e.g., antibodies) conjugated with a colorimetric indicator (e.g., gold nanoparticles = AuNPs) to detect a range of targets such as parasites, bacteria, viruses, genetic modification in crops, and even antibiotics [35,36,37,38,39,40,41,42,43,44]. The solution is migrated spontaneously via capillary action, and the tagged amplicons + bioreceptor + colorimetric indicator complexes are captured and accumulated on the test line of a strip, where the bioreceptor is immobilized. Unbound colorimetric indicators gather on the control line. Aside from tagged probes/primers, Cas enzymes (12a and 13a) have also been used with LFIA strips to detect *Magnaporthe oryzae* (SDH1 and TEF1 genes), GMO (Cry1c gene), *Toxoplasma gondii*, *Verticillium dahliae*, and DNA methylation (SEPT9 gene) [45,46,47,48]. For this detection system, guide RNA (complementary to the target) and reporter probes were added to the DNA sample. Specifically, the template would bind to the target causing the activation of Cas12a or 13a enzymes to perform nonspecific trans cleavage in the unbound regions of the reporter. This action then allowed reporter probes to generate the detection signal. In the LFIA CRISPR/Cas system illustrated in Figure 3 (top left), the ssDNA was modified with biotin and 6-carboxyfluorescein (FAM). Detection follows a standardized scheme, with immobilized anti-IgG on the test line and streptavidin on the control line. The anti-IgG captured the anti-FAM conjugated AuNPs bound with the FAM tag on cleaved reporters, while the streptavidin bound with the biotin tag that remained on uncleaved reporters.

In addition to colorimetric detection, other methods have been coupled with RPA, including surface-enhanced Raman spectroscopy (SERS), fluorometry, and photoelectrochemical detection. For the SERS RPA assays, specialized gold nanoprobes containing 4-mercaptobenzoic acid (4-MBA) were used, with 4-MBA acting as a signaling molecule to detect *Salmonella* Typhimurium, as depicted in Figure 3 (top right) [49]. Linker DNA crosslinked these SERS nanoprobes. Upon successful amplification, the linker DNA was cleaved by the Cas12A enzyme, resulting in a colloidal solution that maintained an even distribution of nanoprobes even after mild centrifugation. In contrast, when the target was missing, the nanoprobes remained crosslinked and were easily aggregated. This change resulted in differing SERS signals, with the no-target solution having lower SERS intensity. For the fluorometric RPA assays, probes tagged with FAM were used for fluorescent labeling and subsequent detection of HIV-1, SARS-CoV-2, and *Helicobacter pylori* [50,51]. The fluorescent signal increased as the target amplicon concentration increased, enabling target detection. Lastly, photoelectrochemical (PEC) detection involved primers tagged with Bi_2_S_3_, which acted as a photoanode, as depicted in Figure 3 (bottom left) [52]. Screen-printed paper-based electrode (SPPE) surfaces were functionalized with photocathodes, specifically AuNPs or Cu_2_O nanocubes, for detecting *Escherichia coli* O157:H7 and *Staphylococcus aureus*, respectively. When the target was successfully amplified, the resulting Bi_2_S_3_ amplicons were immobilized on the SPPE, enhancing PEC signal output. [52]. The fluorescent signal increased as the target amplicon concentration increased, enabling the detection of HIV-1, SARS-CoV-2, and *H. pylori*, respectively.

Of the amplification methods and articles included in this review, RPA is the only method with assays of every category (extraction, amplification + detection, detection, and fully integrated). Colorimetric, SERS, fluorometric, and PEC detection methods have all shown the capability to be combined with amplification into a single device [44,50,52]. In the fluorometric and PEC examples, RPA reagents were added to a paper-based platform that was subsequently heated for reaction incubation. Amplification was then followed by detection on the same paper membrane. The colorimetric assay was slightly different, as the amplification was carried out in a separate reaction chamber, followed by the independent LFIA strip detection. One example showed a fully integrated RPA assay, incorporating target extraction on top of amplification and fluorometric detection, which will be further discussed in Section 7 [51]. However, the only paper-based portion of this assay was the target extraction. It first involved capturing the target using an FTA^®^ card (cotton-based cellulose paper) binding disc and washing it with ethanol. The binding disk containing the captured target was added to a PMMA (polymethyl methacrylate) reaction chip. There are also RPA examples where paper-based extraction is not fully integrated into an assay or performed separately from amplification and detection. Most of these examples involved pretreating samples with lysis or extraction buffer with subsequent target extraction using Whatman^®^ grade 1 filter paper, followed by washing buffer [45,53,54]. This extraction method has been utilized to detect *Magnaporthe oryzae*, GMO, *Toxoplasma gondii*, and various potato viruses, including potato leafroll virus and potato virus S. Another example is where the target was extracted via a Whatman grade GF/F glass microfiber filter embedded in a paper chip. After the sample was dispensed onto the chip, it was soaked in a lysis buffer followed by magnetic bead purification [46]. 

## 3. RAA and MIRA with Paper Microfluidics

Recombinase-aided amplification (RAA) is similar to RPA, but this method uses slightly different enzymes and operating temperatures. RAA generally utilizes four enzymes: UvsX, UvsY, SSB, and polymerase, which displace and bind primers to the template DNA. Initially, a recombinase binds and forms a nucleoprotein complex with the forward primer and the reverse primer. The newly synthesized nucleoprotein complex recognizes and hybridizes with homologous DNA sequences within the template dsDNA. This action creates the template ssDNA, while the homologous sequence is displaced. Complementary strands are then generated from the ssDNA, where the single-stranded binding protein (SSB) binds to the ssDNA, and polymerase binds to the primer–DNA complex. Additionally, amplification occurs at a single temperature between 37–42 °C and within a short reaction time of 10–30 min [55,56]. This amplification method is advantageous over other isothermal amplification methods because of its simplicity, rapid amplification time, high sensitivity, and high specificity. One article compared the sensitivity and specificity of RAA for various pathogens and discovered that the sensitivity ranged from 95% to 100%, while the specificity was between 98.7% and 100% [57]. Due to these advantages, paper-based RAA devices have become increasingly popular, as the number of paper-based RAA publications has steadily increased (Figure 1). Of the ten paper-based RAA articles incorporated in this review, all reported paper-based detection but did not show paper-based extraction or amplification. Nine articles reported colorimetric detection using tagged probes/primers and AuNPs [58,59,60,61,62,63,64]. For example, Xu et al. used LFIA to detect *Amphidinium carterae* by implementing a species-specific primer tagged with biotin and a probe labeled with FAM [62]. In a few cases, Cas12a reporter probes and AuNPs were implemented for detection [65,66]. All colorimetric paper-based RAA detection articles utilized LFIA, explaining why most articles reported a similar detection scheme. In one example, the Cas12a detection scheme was incorporated into a flow distance platform that could be quantified using a simple ruler, as depicted in Figure 3 (bottom right) [67]. Specifically, Cas 12a was used to detect and cleave linker DNA incorporated into a hydrogel. Cas 12a cleavage was only activated when the target DNA was successfully amplified. Cleavage of the linker DNA led to the subsequent release of glucoamylase, which acted as a signaling molecule. Downstream modulation of glucose levels affected the flow distance, with higher glucose concentrations positively related to flow distance. 

Another derivative of RPA is multienzyme isothermal rapid amplification (MIRA). The amplification mechanism of MIRA uses helicase and recombinase to generate ssDNA and initiate the amplification reaction [68]. After reverse transcription, the recombinase complex binds the primer, and the DNA is opened with a single-strand binding protein (SSB), so the primer with the recombinase can bind. This event occurs at both the forward and reverse end of the DNA. Once the primer recombinase complex binds to the ssDNA, DNA polymerase synthesizes the new complementary DNA strand [69]. The use of helicase (gp41) and recombinases (*Streptomyces coelicolor* recA, SC-recA) allows for rapid amplification that can happen between 10–30 min at a constant temperature between 35–42 °C [68,69]. MIRA has also been demonstrated with paper strips and paper microfluidic platforms [68,69,70,71,72,73,74,75]. Like the RAA, all paper-based MIRA assays utilized LFIA with colorimetric detection. They all used tagged probes/primers and AuNPs. In most cases, the primer was tagged with biotin, and the probe was tagged with FAM. Unlike RAA, one article demonstrated paper-based extraction with MIRA to detect *Streptococcus agalactiae* in milk samples [70]. A two-minute disposable extraction was accomplished by adding the sample to a lysing buffer, followed by using a disposable extraction device (DED), consisting of a syringe attached to a filter paper, to mix and aspirate the pre-lysed sample in an elution buffer [70]. In the end, the filter paper was washed and used for the subsequent amplification assay.

Figure 3 summarizes three paper-based detection examples with RPA (colorimetric, SERS, and photoelectrochemical) and one flow distance-based detection example with RAA.

## 4. LAMP with Paper Microfluidics

Loop-mediated isothermal amplification (LAMP) is a method that utilizes DNA polymerase along with four to six primers to amplify nucleic acid sequences. This technique is initialized by DNA polymerase for strand displacement of the target DNA [76]. The target DNA has six regions known as F3c, F2C, F1c, B1, B2, and B3 that can bind to the LAMP primers, where F denotes forward, and B represents backward. The three forward regions are upstream of the target DNA sequence, while the backward regions are downstream. After strand displacement, the forward inner primer (FIP) binds to the complementary F2c region of the target DNA. The binding of FIP at the 3′-end of the target sequence initiates the synthesis of the complementary DNA strand. Another primer, the forward outer primer (FOP), binds to the complementary F3c region of the target sequence. The binding of these primers causes displacement of the F1c region created from the FIP, so the F1c region now binds to the F1 region of the complementary strand. This process creates a looped structure on the 5′-end of the complementary strand where the F1 and F1c regions are aligned to interact with each other, and the F2 region is the area that makes up the loop. A similar process is repeated on the 5′-end of the target, and the resulting product has a dumbbell shape. The product at the end of this amplification is a strand of DNA with multiple loops and lengths. The LAMP process is depicted in Figure 2 in the middle. LAMP is typically performed using a thermal cycler at 65 °C and allows for diverse detection methods, including colorimetric, fluorometric, gel electrophoresis, and turbidity detection [77,78].

**Figure 4:**
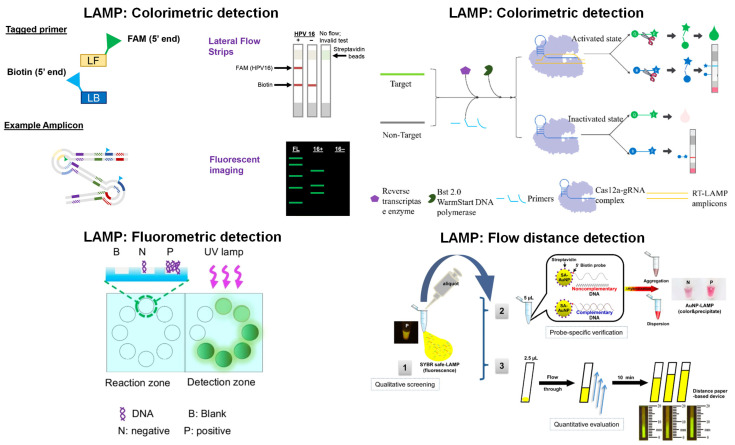
Paper-based detection examples with LAMP. In the colorimetric detection (**top left**), the primers tagging the 5′-end allow for amplification without inhibition of tags. LFIA strips with fluorescent gels characterize the LAMP products when the FAM tag attaches to the anti-FAM test line. In the other colorimetric detection (**top right**), primers perform reverse transcription amplification of genes. When the Cas12a-gRNA complex is activated, ssDNA is cleaved, which can be modified with a fluorescent and quenching group. Once separated, the fluorescence is observed with 480 nm light. In fluorometric detection (**bottom left**), fluorescence signals from the reaction and detection zones are compared, and the values are displayed on the smartphone screen. In the flow distance detection (**bottom right**), fluorescent SYBR-quantified LAMP products were conjugated with AuNPs on paper. Using DNA-immobilized cellulose, qualitative screening with a blue light illuminator evaluates the fluorescent migratory distance for the amounts of LAMP product. Reprinted with permissions from [81], Copyright 2020 Springer Nature; [96], Copyright 2022 American Chemical Society; and [89], Copyright 2022 American Chemical Society. Reprinted from [84] under Creative Commons Attribution License. Note: [89] demonstrated amplification in addition to detection, and [96] showcased a fully integrated system.

Paper-based detection, amplification + detection, and fully integrated systems have been demonstrated with LAMP. While detection is still the most widely used application of paper microfluidics for LAMP, amplification + detection and fully integrated systems have also been extensively investigated. The most common type of detection for paper-based LAMP assays is fluorometric, which differs from a conclusion drawn from [31], where the authors suggest that colorimetric is a better detection method since fluorescent dyes can inhibit the LAMP reaction. That review analyzed papers from 2015 to 2021, whereas this paper analyzed those from 2020 to 2023. Fluorescent dye was used as the sensing element for seven key LAMP references, indicating that florescent dyes are becoming more popular for detection. The most common type of detection was colorimetric, with the most basic detection systems involving a standard LFIA, where amplified LAMP products are loaded onto the paper test strips [79,80,81,82]. Additionally, these sensing systems involved oligonucleotides modified with biotin, FAM, fluorescein, and FITC. For example, the system developed by [81] utilized LFIA strips to detect HPV 16 for POC diagnostics, as depicted in Figure 4 (top left). In this work, LAMP was performed using a traditional thermal cycler at 63 °C for 60 min, and the products were added to the LFIA strip’s sample pad, with detection occurring within 5 min. Specifically, this work utilized an anti-FAM antibody on the test line and a biotin antibody on the control line of the strip. Lateral flow is not the only colorimetric detection method that has been demonstrated with LAMP products. One article implemented a primer tagged with eosin dye to detect *Mycobacterium tuberculosis*. In this system, the amplified product was loaded onto streptavidin-conjugated cellulose paper, and detection was executed using green LED to illuminate the paper and a smartphone camera to capture the colorimetric signal intensity [83]. Ruang-Areerate et al. [84] demonstrated a fluorescence-distance-based LAMP system to detect *Leishmania* in asymptomatic patients with *Leishmania*/HIV co-infection, as depicted in Figure 4 (bottom right). They created a single-step assay using AuNP probes and SYBR Green fluorescent dye to enhance specificity and sensitivity. Samples were loaded onto the custom-designed, wax-printed paper microfluidic channels flowing from the inlet. Fluorescence could be observed until the amplicons were depleted, and they measured the distance of such fluorescence (under blue excitation) in millimeters.

Paper-based detection systems have been incorporated with amplification performed on paper [85,86,87,88,89,90]. One example included the real-time fluorescence measurement of the amplified products on a paper microfluidic chip to detect *Escherichia coli* and *Campylobacter jejuni* [85]. This device utilized inexpensive components such as a CMOS camera, a microcontroller, a thermocouple, and a thermoelectric cooler. Most paper microfluidic amplification + detection systems with LAMP involved layered paper microfluidic chips, with a sample loading chamber, a washing layer or purification layer, and a reaction chamber preloaded with the LAMP reagents. Then, a target DNA or RNA sequence was loaded into the sample inlet, where the LAMP reaction flowed into the reaction chambers where amplification occurred [85,86,88,89,90,91,92]. These samples flowed through the paper microfluidic chambers by puncturing the protective layer of film and flipping the microfluidic chip over or by a series of origami-like folds to move the sample from one region to another. The constant temperature between 60–65 °C was maintained using various techniques, such as a hot plate or a heat block.

In some applications, detection was also executed during amplification, e.g., real-time detection. The real-time detection allowed the generation of amplification curves, which are beneficial for monitoring, optimizing, determining the earliest detection time, and obtaining quantitative data. If real-time detection was not implemented, qualitative or semi-qualitative detection was performed by visual inspection or by obtaining images and subsequent image processing. For example, Choopara et al. [87] presented a paper-based LAMP amplification and fluorescence detection system to detect methicillin-resistant *Staphylococcus aureus* (MRSA). Amplicons were fluorescently visualized with SYBR Green dye, using a UV transilluminator for excitation and a smartphone for acquiring fluorescent images. These images were then processed using ImageJ to collect the intensity values. Many paper-based LAMP amplification and detection systems have utilized fluorescence detection with intercalating dyes such as SYBR Green or fluorescent probes incorporated into the LAMP reaction. In some cases, calcein and Mn^2+^ were used as the fluorescence sensing mechanism. Additionally, some studies have coupled Cas12a enzymes with fluorescent ssDNA probes for detecting the amplified LAMP product. Sen et al. [90] demonstrated this technique, which enhanced the sensitivity and specificity of LAMP by incorporating CRISPR/Cas technology to detect *Escherichia coli*. 

Paper-based LAMP has also been carried out as a fully integrated system, which performed extraction, amplification, and detection on paper. DNA or RNA extraction is the most challenging aspect of isothermal amplification to perform on paper due to the difficulty of integrating extraction techniques with the subsequent amplification system. Paper-based extraction leads to the dilution of the extracted nucleic acids and increases the likelihood of low detection sensitivity [93]. However, despite these challenges, a few studies have developed fully integrated paper-based LAMP devices. Most of these methods used a fluorometric detection system as exemplified in Figure 4 (top right and bottom left), with DNA extraction being carried out with either an FTA card or a lysis buffer [85,87,92,94,95,96]. FTA cards were manufactured from cellulose paper and had reagents immobilized on the paper, which prompted cell lysis and protein denaturation. Additionally, they stabilized the released nucleic acids to be stored and preserved for further processing [97,98]. FTA cards have become popular for the field collection of DNA and RNA due to their low cost, user-friendliness, and minimal storage requirements, as the cards can be stored at room temperature and provide long-term storage [97,98]. For example, the assay developed by [95] utilized this DNA extraction method to detect SARS-CoV-2 and *Enterococcus faecium* in a fully integrated device with colorimetric detection. Extraction involved adding bacteria culture media to the FTA card, followed by incubation. This was preceded by purification and washing with a purification reagent and TE buffer. Other methods for DNA extraction involved using a lysis buffer to lyse the DNA, which was then captured by a paper membrane [85,87,94,96]. For instance, in the integrated system developed by Garneret et al. [94], the collected SARS-CoV-2 target from nasopharyngeal swabs was initially mixed with AVL buffer before being added to the extraction membrane. This design allowed the nucleic acids to be trapped within the membrane while the other cellular components flowed through the membrane. Following the entrapment of the nucleic acids, the device was incubated and washed with ethanol and washing buffer. In terms of amplification, these fully integrated devices used similar mechanisms to those employed by the amplification + detection devices where LAMP reagents were dried onto paper substrates, and the newly extracted DNA was transferred to the reaction pad; then, the device was incubated for amplification, and fluorescence detection was performed. These fully integrated devices are beneficial for performing POC diagnostics in low-resource settings since they offer a low-cost alternative to traditional LAMP protocols, eliminating the need for complicated DNA extraction methods while maintaining the same level of specificity and sensitivity.

## 5. RCA with Paper Microfluidics

As the name suggests, rolling circle amplification (RCA) is an amplification method that uses strand displacement polymerase to amplify circular template DNA. This circular template DNA contains a single-stranded region where the amplification process is initiated. A primer complementary to the template strand is then introduced and anneals with the template strand. Moreover, strand displacement polymerase creates a new complementary strand, and this newly synthesized strand displaces the original single-stranded region of the template, thus creating a new single-stranded region for the following primer to bind [99,100]. The RCA process is depicted in Figure 2 on the right. This amplification process is exponential, thus yielding high amounts of DNA within a short time [101]. Additionally, this method can be used for detecting a myriad of nucleic acids, including single nucleotide polymorphisms (SNPs), DNA aptamers, DNAzymes, spacer domains, and restriction enzyme sites [102]. Since this is an isothermal amplification method, it is performed at a constant temperature, ranging from room temperature to 37 °C, simplifying the entire amplification process by eliminating the need for an expensive heating element. Other advantages of RCA include high sensitivity, where exponential RCA can reach 10^9^-fold, and high specificity since RCA can detect SNPs [103].

**Figure 5:**
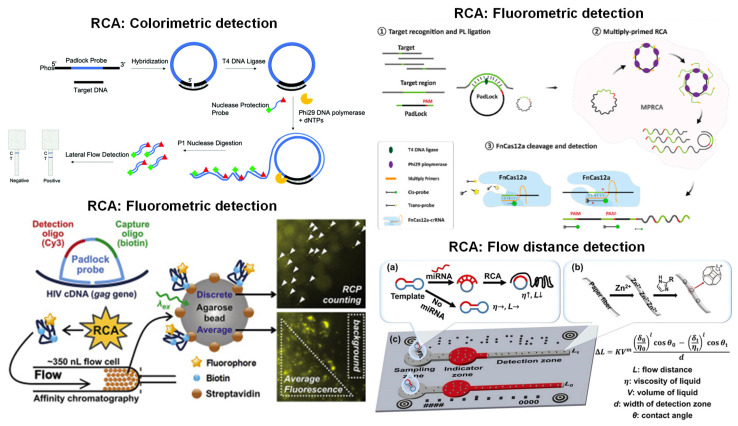
Paper-based detection examples with RCA. Colorimetric detection (**top left**) exposes the sample to a nuclease, which cleaves a selected ssDNA fragment detected in an LFIA strip. In fluorometric detection (**top right**), the target RNA is ligated using the padlock probe with a complementary sequence of the target RNA virus and a DNA ligase. The FnCas12a/crRNA complex cleaves targeted dsDNA regions, which emit fluorescence. The nonspecific ssDNA is also detected from a fluorescence reader. In the other fluorometric detection (**bottom left**), the RCA products are pre-labeled with biotin and fluorophore, efficiently capturing the fluorescence signal at high resolution and sensitivity. In distance detection (**bottom right**), the microfluidic paper-based analytical devices (μPADs) contain indicator and detection zones to quantify miRNAs via viscosity amplifications. The pre-amplified miRNA (a) is placed in the sample well, and the difference in the target and control flow are enhanced with surface hydrophobicity modulations (b). It is paired with smartphone auto-reading systems to improve accuracy (c). Reprinted with permissions from [106], Copyright 2021 Royal Society of Chemistry; [111], Copyright 2023 Elsevier; [114], Copyright 2020 Elsevier; and [113], Copyright 2022 Elsevier.

Most of the paper-based RCA systems were designed chiefly for detection. Various detection techniques were demonstrated for paper-based RCA, including colorimetric, fluorometric, and flow distance. However, most articles featured colorimetric methods since LFIA was mainly used for product detection [104,105,106,107,108,109,110,111,112], where probes or primers were tagged with AuNPs, biotin, or FAM modifiers [104,105,107,109]. This sensing system with RCA amplification has been used to detect a wide range of targets from *Chattonella marina*, *Prorocentrum minimum*, methicillin-resistant *Staphylococcus aureus* (mecA gene), and DNA methylation of GlaI digestion products. The assay developed by [107] applied LFIA to detect the mecA gene after RCA. The RCA amplification used a padlock probe specific to the target DNA with an additional biotin primer and FAM probe. Streptavidin and anti-FAM were immobilized on the LFIA strip. While the tagged probes/primers with AuNPs appear to be the most widely used LFIA technique, other studies have used alternative probes and receptors. For example, Li et al. [108] analyzed salivary miRNA-31 by LFIA, where detection relied on tagged hairpin probes and hemin-mediated TMB oxidation. Another sensing element for RCA + LFIA detection is a padlock probe along with biotin- and digoxigenin-modified nuclease protection probes to recognize the SARS-CoV-2 beta-lactamase resistance gene, illustrated in Figure 5 (top left) [106].

Only one article reported the use of flow distance on paper for detection after RCA, depicted in Figure 5 (bottom right) [113]. Flow distance is beneficial since quantitative data can be obtained by measuring the flow distance within each microfluidic channel. The RCA amplicons from miR-221 and miR-222 were loaded into the sample zone, which flowed into the indicator zones through capillary action. Within the indicator zone resided the red ink. The amplified sample entered the indicator zone, mixed with the red ink, and continued flowing into the detection zone. The detection zone was modified with metal-organic frameworks (MOFs) to modulate the paper’s hydrophobicity. With increasing RCA amplicons, the sample’s viscosity increased, and the flow on the hydrophobic channel was shortened. The detection was observed via naked-eye inspection or smartphone imaging and analysis.

Fluorometric detection was the second most used technique in paper-based RCA detection [110,114,115]. In these works, a probe was tagged with a fluorescent signaling molecule, such as quantum dots, Cy3, or microspheres. For instance, Liu et al. [115] carried out fluorescence detection of miR-21 and circRNA-HIAT1 on cellulose paper modified with citric acid. This system formed blue-emissive carbon dots when carbonized. When the DNA with the fluorescent label and RCA product hybridized, red or green fluorescence was generated, and the fluorescence of the blue carbon dots was quenched. The fluorescence signal was measured by capturing smartphone images in a dark room with UV light excitation, and RGB intensity values were extracted from the images. Soares et al. [114] also demonstrated fluorescent detection with Cy3 for detection of HIV-1, as depicted in Figure 5 (bottom left). 

While most paper-based RCA demonstrated only detection, two articles demonstrated both amplification and detection [116,117]. Both works utilized fluorometric detection with intercalating dyes, including SYBR Green and QuantiFluor dye. In the first publication [116], test areas were wax printed onto a nitrocellulose membrane, and a blank film was created by printing and drying pullulan solution onto the paper. Next, the RCA reagents, including padlock probe, primer 1, primer 2, T4 DNA ligase, and SYBR Green I, were added to create the bioactive film. The target DNA was added to the nitrocellulose paper, and the device was incubated at 30 °C for 240 min for amplification. Upon reaction completion, fluorescence was measured using a multi-mode microplate reader with an excitation wavelength of 490 nm to detect SARS-CoV-2. The second article [117] demonstrated paper-based amplification and detection to detect platelet-derived growth factor (PDGF) and thrombin on microwell-patterned, wax-printed cellulose paper. The cellulose paper was treated with pullulan-encapsulated RCA reagents and was dried overnight. Buffer, QuantiFluor dye, primer, and target were loaded onto the cellulose paper for amplification. The paper was incubated at room temperature, and real-time fluorescence was measured from a gel documentation system. These two articles demonstrate that fluorescence detection for paper-based RCA could be achieved with a microplate reader and image processing. 

Paper-based extraction or fully integrated devices have rarely been demonstrated with RCA for 2020–present. Only one work was reported in 2019 (outside this review period): Sun et al. [118] fabricated a paper microfluidic chip containing five panels made from chromatography, filter and nitrocellulose paper, and an absorbent pad. Each panel performed a specific function, including sample purification and washing, cell lysis, DNAzyme recognition, and amplification.

Overall, paper-based RCA has been successfully demonstrated mostly for detection, with two works showing amplification + detection and one showcasing a fully integrated system. The detection time ranged from 2 min to 180 min.

## 6. Other Isothermal Amplification Tests with Paper Microfluidics

Other isothermal amplification tests have also been implemented on paper-based testing platforms, such as nucleic acid sequence-based amplification (NASBA), helicase-dependent amplification (HDA), strand displacement amplification (SDA), exponential amplification reaction (EXPAR), and competitive annealing mediated isothermal amplification (CAMP). Of these amplification methods, NASBA and HDA appear to be the more popular choices to integrate with paper microfluidics, as shown in Figure 1. The next most popular method is SDA, while EXPAR and CAMP are minimally integrated with paper microfluidics. Like the other isothermal amplification tests described in the previous sections, paper-based detection is the most used application for all five isothermal tests. Specifically, NASBA and HDA were exclusively demonstrated with paper-based detection, while SDA and EXPAR were also incorporated with paper-based amplification. For example, paper-based amplification and detection were published in 2022 with SDA and 2021 with EXPAR [119,120]. One publication reported paper-based extraction with CAMP [121]. This is also the only CAMP article included in this review. Furthermore, only one article reported a fully integrated device with EXPAR [92].

**Figure 6:**
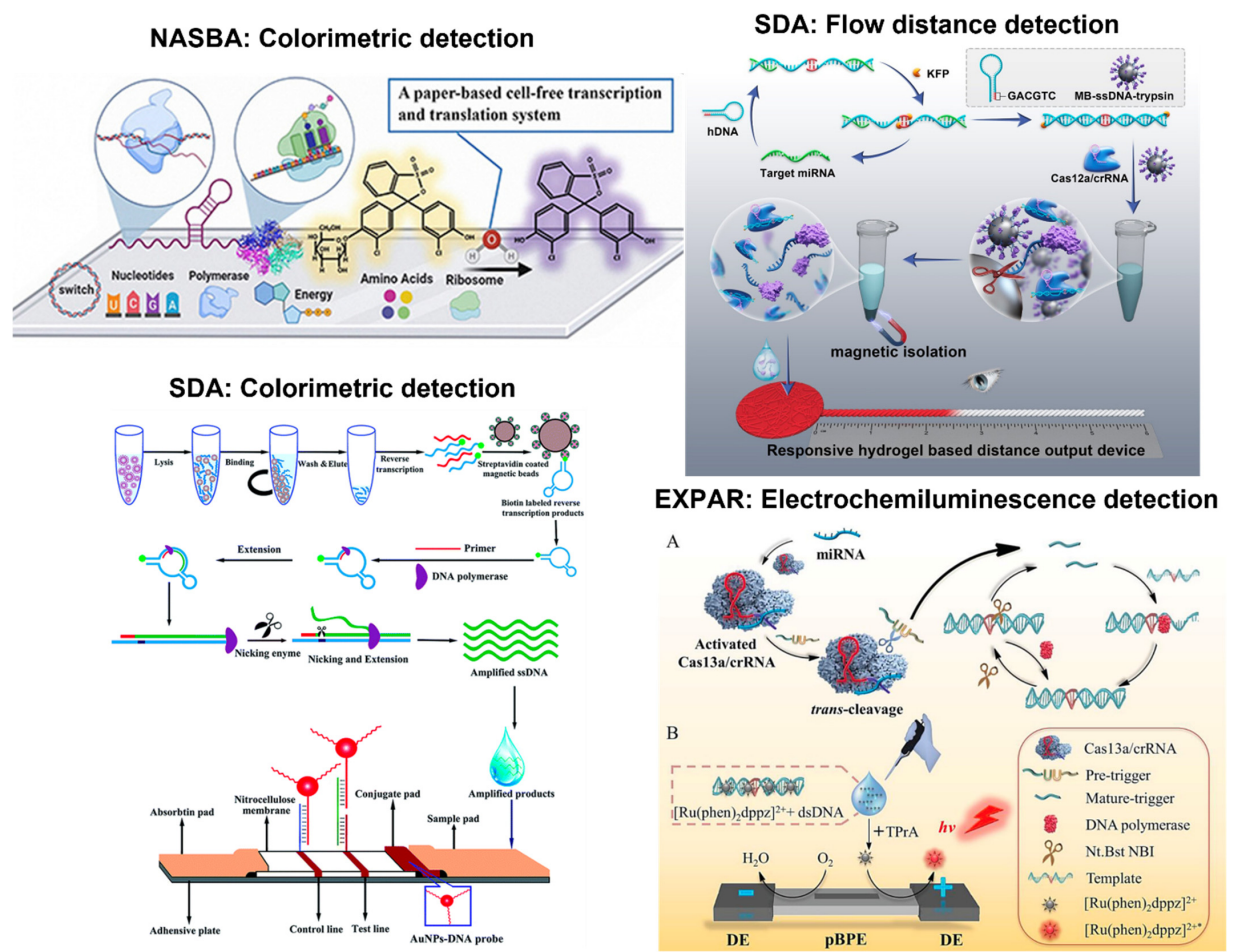
Paper-based detection examples with NASBA, SDA, and EXPAR. NASBA (**top left**): colorimetric detection is used to amplify the target RNAs to produce a visible signal that is sensitive and specific to differentiate RSV subgroups within the paper-based system. SDA (**top right**): flow distance detection is used, where the target miRNA is converted into dsDNA that triggers CRISPR/Cas12a cleavage activity to release trypsin. The release of trypsin hydrolyzes gelatin to increase permeability and increase the signal. SDA (**bottom left**): hemorrhagic fever viruses can be rapidly detected using magnetic beads and nicking enzyme-assisted isothermal strand displacement amplification. The nucleic acid sequence is amplified with a cleavage enzyme and a polymerase that extends the sequence. Once obtained, the amplified DNA is loaded on the sample pad for visual detection on the LFIA strip. EXPAR (**bottom right**): specific and sensitive miRNA detection can be performed using CRISPR/Cas13a, such that this system recognizes the target miRNA to initiate cleavage activity. The cleavage produces two fragments that hybridize with the template and form dsDNA that undergoes strand extension to generate more dsDNA products (A). These products can interact with the [Ru(phen)_2_dppz]^2+^ ligand to increase the luminescence collected through the photomultiplier tube (B). Reprinted with permissions from [123], Copyright 2021 Elsevier; [133], Copyright 2023 American Chemical Society; and [130], Copyright 2020 Royal Society of Chemistry. Reprinted from [139] under Creative Commons Attribution License.

Like the other isothermal amplification tests, colorimetric detection was the most commonly demonstrated method on the paper platforms, e.g., all NASBA references reported colorimetric detection. Three works incorporated a toehold switch and β-galactosidase-mediated chlorophenol red-β-D-galactopyranoside cleavage into the detection system for the identification of norovirus and respiratory syncytial virus, and in another system for the Zika and Chikungunya viruses [122,123,124]. In this arrangement, the toehold switch regulated the synthesis of β-galactosidase and was designed to have a region complementary to the target sequence. When the toehold switch encountered the target sequence, the strong ribosome binding site would initiate the synthesis of β-galactosidase (LacZ). In the presence of chlorophenol red-β-D-galactopyranoside, LacZ would cleave the compound to yield chlorophenol red, causing red-β-D-galactopyranoside to change its color from yellow to purple, which could be observed by visual inspection. This mechanism is illustrated in Figure 6 (top left). An alternative sensing system with NASBA included tagged probes and neutravidin-conjugated carbon nanoparticles (NA-CNPs) for detecting SARS-CoV-2 [125]. This scheme follows standard LFIA detection using biotin and FAM-modified oligonucleotide with AuNPs, where NA-CNPs replaced AuNPs. The dual-tagged RNA allowed the FAM end of the RNA to bind to the anti-FAM antibody on the test line while also leading to the aggregation of NA-CNPs to create a visible detection line. The third sensing system utilized biotin-modified oligonucleotides and AuNPs to detect *Macrobrachium rosenbergii* nodavirus [126]. Instead of using FAM, a FITC-labeled DNA probe was used, and monoclonal anti-FITC antibody was placed on the test line while anti-biotin antibody was placed on the control line.

This LFIA detection system can also be seen with HDA to identify SARS-CoV-2 [127]. Alternatively, biotin and FAM-modified oligonucleotides with AuNPs were employed for hepatitis B virus screening [128]. HDA and LFIA sensing systems were also used to diagnose SARS-CoV-2, utilizing streptavidin-coated blue latex beads and biotin [129]. For this assay, a tag linker sequence and biotin-labeled primer bound to streptavidin-coated blue latex beads, which colored the target sequences blue. A probe, complementary to the tag linker, was embedded in the paper strip for detection.

All SDA work demonstrated colorimetric LFIA detection using tagged probes and AuNPs [130,131,132] (One example is shown in Figure 6, bottom left). Additionally, one flow distance-based detection system utilized gelatin-treated paper permeability via Cas12a-mediated trypsin release to recognize miRNA-let-7a, which is highlighted in Figure 6 (top right) [133]. Upon successful amplification, Cas 12a cleavage was activated to cleave ssDNA that conjugated trypsin to magnetic beads. When the trypsin was removed from the magnetic beads, it could degrade the gelatin in the sample pad, modulating permeability and eventually affecting flow distance. Specifically, higher target concentration led to greater trypsin release, increased permeability, and longer flow distance. Another work demonstrated a paper-based SDA amplification and fluorometric detection system to identify methicillin-resistant *Staphylococcus aureus* (MRSA) [119]. They used a paper membrane with lyophilized SDA amplification reagents and fluorophore-labeled probes. The device was designed to segment each image so that each glass fiber amplification pad represents approximately 5 × 10^4^ to 10^5^ pixels to reduce the noise-to-signal ratio.

Three papers reported paper-based colorimetric detection systems with EXPAR. All used tagged reporter probes and AuNPs to distinguish the presence of various targets. One article exemplified using a tagged TtAgo reporter probe with AuNPs [134]. Argonaute proteins (Agos) are endonucleases capable of cleaving DNA and RNA. Agos can use DNA to find the target, unlike CRISPR/Cas systems that use guide RNA and protospacer-adjacent motifs (PAM) [135]. The general mechanism of DNA-guided Argonaute from *Thermus thermophilus* (TtAgo) is as follows: the TtAgo binds to the guide oligonucleotides, and this complex then searches for the complementary target sequences. When the guide binds to the target sequence, an endonuclease is activated to cleave complementary strands [136]. The mechanism was coupled with LFIA in this demonstration, where the FAM–biotin reporter probe was complementary to the TtAgo EXPAR amplified product [134]. The FAM–biotin reporter probe allowed for a standard LFIA to be performed, where the probe was disrupted in the presence of the target complex, thus leading to the aggregation of AuNPs in the test zone. Without the target, the aggregation appeared solely in the control zone. The other probes used with AuNPs for EXPAR LFIA were hairpin probes tagged with either biotin or digoxin at the 3′ end to indicate the presence of nerve growth factor-beta, as well as the probes tagged with FAM and biotin for the detection of telomerase activity (more specifically, telomerase elongation products from human cancer cell lines) [137,138]. Electrochemiluminescence detection was also coupled with EXPAR, where a Cas13a reporter probe along with ruthenium complex [Ru(phen)_2_dppz]^2+^ tag oxidation was used to detect miR-17, which is depicted in Figure 6 (bottom right) [139]. Similar to the previously described CRISPR/Cas 12a system, the CRISPR/Cas13a mechanism functioned similarly where a guide RNA was designed to be complementary to the target. When the guide RNA encountered the target, Cas13 randomly cleaved nearby RNA that included the reporters [140,141]. In this scenario, the ruthenium complex served as a light switch due to its luminescence property being amplified in the presence of DNA. The luminescence signal was then measured through a photomultiplier tube (PMT) with the driving voltage supplied by DC power.

Despite detection being the most commonly used paper application of the EXPAR assays, there has been an attempt to perform amplification and fluorescence detection on paper with EXPAR to detect miRNA-223 [120]. In this layout, the paper-based miRNA sensor consisted of five layers, with the top and bottom layers being made from PCR adhesive film, the second PES channel layer serving as the sample inlet, the third layer consisting of six glass microfiber reaction pads, and the fourth a silicone frame layer. The general workflow was similar to other amplification and detection systems, where the template, EXPAR regents, and SYBR Green dye were loaded into the inlet chamber. They flowed through the PES channel to the reaction pads, where miRNA was dried and stored. The amplification was performed at 58 °C for one hour, while real-time fluorescence images were captured in 30-s intervals.

The last isothermal amplification method included in this review is CAMP, but there is a minimal number of paper-based applications with this method. However, one recent publication showed paper-based extraction of *Shigella* DNA using a similar protocol to the one previously described within MIRA, where pre-lysed samples were passed through a syringe containing filter paper [121].

## 7. Discussion

There have been many isothermal amplification methods that have been implemented with paper microfluidics. These amplification mechanisms vary by the number of primers, operating temperature, and amplification time. LAMP and SDA require the most primers, where LAMP uses four to six primers, and SDA uses four [76,142]. RPA, RAA, MIRA, and CAMP use two primers, while RCA, NASBA, HDA, and EXPAR are executed with a single primer [69,143,144,145,146,147]. Temperature is another factor that makes some paper-based amplification methods better suited for low-resource settings or fieldwork. The ten amplification methods investigated in this review can be categorized into low or high amplification temperatures. LAMP, HDA, SDA, EXPAR, and CAMP are performed at high temperatures ranging from 60–65 °C [76,142,143,146,147]. RPA, RAA, MIRA, RCA, and NASBA have low amplification temperatures between 20–42 °C [55,68,103,148]. These amplification methods can also be sorted by total assay time. It is challenging to generalize the reaction time since each assay is designed differently. However, MIRA appears to have the fastest average total assay time of 18.75 min [68,70,72,74]. One review paper summarized the same characteristics of various isothermal amplification methods. In summary, RPA has the shortest reaction time between 5-7 min and only uses one primer [149]. The quick reaction time and simplicity of only having one primer could be one reason why RPA is a popular choice for paper-based amplification. Other comparisons of isothermal methods recognize that HDA and NASBA also average the same time of approximately 1.5 h for nucleic acid amplification and use only one pair of primers for these isothermal methods, but NASBA can be utilized with a larger variety of templates. According to this review paper [149], NASBA targets can include ssDNA, transfer-messenger RNA, and ribosomal RNA, making it an amplification method worth further exploration.

Another way to compare amplification methods is by analytical sensitivity or limit of detection (LOD), which is also challenging, as the LOD significantly varies according to the target and detection method. For example, when detecting SARS-CoV-2 with paper microfluidic isothermal amplification tests, one colorimetric detection assay with RPA yielded a LOD of 20 copies/μL, while another colorimetric RPA assay reported LODs of 1 and 0.77 copies/μL for the delta and omicron variants, respectively [38,40]. This result suggests a wide variation in analytical sensitivity or LOD within the same amplification method. As such, comparing the different amplification methods by analytical sensitivity or LOD may not yield any valuable insight.

Another important criterion is the range of targets. For example, RPA has been demonstrated to detect various targets, including bacteria, viruses, fungi, genetically modified crops, DNA methylation, and even antibiotics. Multiplex detection for such a wide range of targets would be helpful in clinical and field settings where testing for multiple targets may be required. Multiplex detection is the ability to detect multiple targets simultaneously within one paper microfluidic device. It is made possible through the several functionalities of paper microfluidics discussed in a 2017 review by Magro et al. [26]. They discussed six functions of paper microfluidics, concluding that multiplex detection resulted from valving, which is defined as the folding of paper to separate, stop, and start fluid flow. Furthermore, multiplexing can be accomplished by paper patterning methods such as wax printing, which creates hydrophobic channels on the paper substrate. Some of the articles within this review developed multiplex detection systems. Of the few multiplex detection articles, half performed fluorescent detection and the other half were colorimetric. LAMP is the most popular amplification method utilized with multiplex detection [85,89,95,96]. Multiplex detection has been used to detect various genes such as SARS-CoV-2 ORF1ab, N, E, and RdRP genes, or multiple different pathogens such as *Escherichia coli* (malB and tuf genes) and *Campylobacter jejuni* (cj0414 gene) [68,85,127]. Another example of multiplex detection of various bacterial species was with the eaeA gene of *E. coli* O157:H7, the invA gene of *Salmonella* spp., and the nuc gene of *S. aureus* [89]. Additionally, multiplex detection has been executed in fully integrated devices with LAMP. In the first fully integrated multiplex system, the esp gene of *E. faecium* and the bla OXA-23-like carbapenemase gene of *A. baumannii* were detected [95]. In the second fully integrated system, S, N, and E genes of SAR-CoV-2 were detected [96]. A recent review on paper-based LAMP explains that more development of multiplex detection would increase the diagnostic ability of paper-based NAATs, thus making them ideal for low-resource clinical settings [31]. A small number of multiplex systems have been developed from the articles incorporated into this review, although it is still a growing area of research. Furthermore, the ability to incorporate multiplex detection into fully integrated systems would make them more suitable for low-resource settings.

Table 1 summarizes all references cited in this review, categorized by the demonstrated procedures: extraction only, amplification + detection, detection only, and fully integrated (all three). Only six articles demonstrated extraction capabilities, and seven showed fully integrated systems that included extraction. This result indicates that paper-based extraction is somewhat under-utilized and has considerable potential for further advancement, which could significantly enable the expansion of fully integrated systems. Paper-based detection is the most-demonstrated process (66 out of 92) incorporated with isothermal amplification tests for 2020–present. This number can be increased to 86 by adding the number for amplification + detection. Specifically, RAA, MIRA, RCA, and many other methods have predominantly been used with paper-based detection. LAMP, followed by RPA, was also used for amplification + detection, extraction, and fully integrated systems. More research should be conducted to demonstrate processes other than detection, especially moving toward development of fully integrated systems.

Table 2 summarizes the various paper-based detection methods. Almost all cited papers reported paper-based detection, including colorimetric, fluorometric, flow distance, SERS, etc. Specifically, RPA was demonstrated with all paper-based detection methods. This result may be significant since a particular isothermal amplification method may require a specific detection method. Such diversity can also be seen with LAMP, RCA, SDA, and EXPAR to lesser extents, while RAA, MIRA, NASBA, and HDA are almost exclusively used with colorimetric detection. Another review paper summarized the advantages and disadvantages of common detection methods [149]. The most notable advantages were low cost, high signal sensitivity, and simplicity across multiple detection methods (electrochemical, colorimetric, chemiluminescence, and fluorescence). The disadvantages differed across the detection methods. Electrochemical detection was noted to have interference with susceptibility and weak stability. Chemiluminescence was reported to be enzyme-dependent and time-consuming. SPR-based detection is real-time and shows a low detection limit, while it requires bulky, costly equipment. Improvement in sensitivity is a significant anticipation for most detection methods summarized in this paper. Furthermore, the integration of systems and increased multifunctionality, while also factoring in biocompatibility and reusability, are also described to make future contributions in their applications, which may be favorable when considering low-resource environments.

## 8. Future Directions and Fully Integrated Systems 

**Figure 7:**
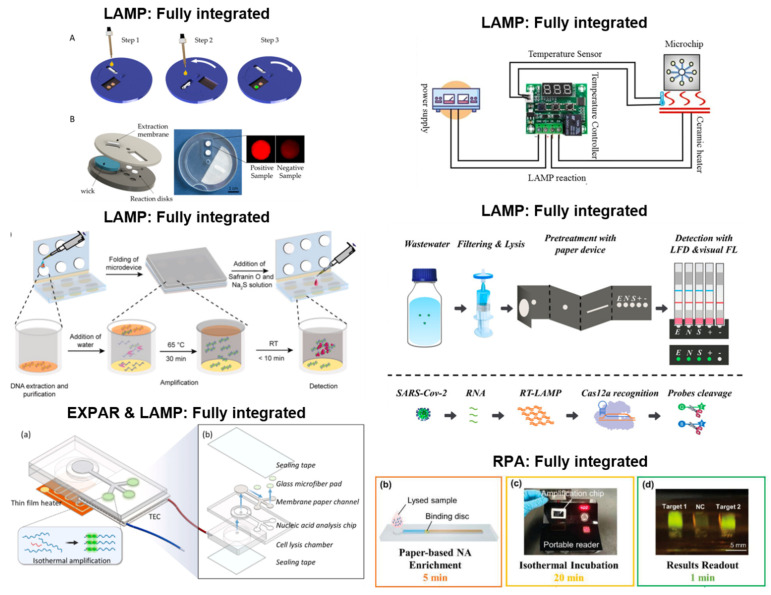
Fully integrated systems with LAMP, EXPAR, and RPA. The fully integrated LAMP (**top left**) comprises three steps, starting with the injection of fluids to the capture membrane and subsequent absorption via wicking (A). Then, the disk rotates and undergoes an elution process before the disk counter-rotates, which induces PCR sealing, amplification, and readout (B). Another LAMP microfluidic device (**top right**) is also pictured, interfaced with a temperature controller and power supply. The third fully integrated LAMP device (**middle left**) combines DNA extraction, purification, amplification, and detection in a microdevice. The bacteria sample is loaded and captured on an FTA card before being introduced to the LAMP reagent chamber, where the DNA target is mixed with the LAMP reagents stored on the disc. After heating the microdevice, the sealant film is removed, and colorimetric detection agents are added. Another fully integrated LAMP system (**middle right**) detects SARS-CoV-2 genes from wastewater samples based on CRISPR/Cas12a-contained base pairs of gRNAs. Fluorescence detection is conducted with probe recognition. EXPAR and LAMP are demonstrated in a fully integrated system (**bottom left**), incorporating a sensor cartridge with a thin-film heater and a thermoelectric cooler for amplification (a). The cartridge layers are expanded to show the individual layers wit channels, chambers, microfiber pads, and analysis chips (b). The heater heats the lysis chamber, which evaporates and absorbs into the paper strip moving to the reaction chamber. The sample is collected on cellulose, where the microfiber filter pad reduces the fluorescence signal. RPA with nucleic acid enrichment on the LFIA strip (**bottom right**) is shown to extract the nucleic acids onto an FTA card (b). The amplification chip is loaded with the FTA card, primers, probes, and RPA reagents and incubated at 40 °C for 20 min (c). The results were observed under 488 nm blue light excitation once the chip was placed into the detection chamber (d). The portable reader visualizes the fluorescence results compatible with smartphone applications. Reprinted from [91,94] under Creative Commons Attribution License. Reprinted with permissions from [95], Copyright 2022 Elsevier; [96], Copyright 2022 American Chemical Society; and [92], Copyright 2023 Elsevier. Reprinted from [51] under Creative Commons Attribution License.

Future development of paper-based NAAT systems will likely be focused on reducing assay time and improving ease of use. As mentioned in the previous section, expanding paper-based extraction will play a vital role. Additionally, fully integrated systems garner special attention as they are ideal for POC scenarios and large-scale testing (Figure 7). They will minimize the time, equipment, and training required for device operation. However, it is essential to make further improvements to reduce the external equipment needed to execute fully integrated systems. Another review article [31] pointed out that many heating elements used for these paper-based isothermal amplification devices could still be expensive, large, and require substantial power. Of the fully integrated devices presented in this review, many of them still used a hotplate or incubator as the heating element; therefore, the development of low-power heating systems could be an area of future study to make them ideal for low-resource settings and field testing. RPA, LAMP, and EXPAR have all been successfully incorporated into fully integrated devices [51,91,92,94,95,96]. Based on the articles included in this review, MIRA, RCA, and SDA have shown great potential for fully integrated systems, as they have already demonstrated paper-based amplification + detection [70,116,117,119]. It should also be noted that LAMP has the highest number of fully integrated assays showcased in this review [91,92,94,95,96].

Notably, some fully integrated assays have additional features that make them even more advantageous. Specifically, the assay developed by [92] can conduct both LAMP and EXPAR. This opens the door for devices capable of performing multiple types of isothermal amplification, allowing users to select the one that best suits their needs. Another example includes a LAMP assay that can perform both fluorometric and colorimetric detection [96]. More to the point, three of the six fully integrated assays presented in this review can detect multiple targets simultaneously [51,95,96]. The level of flexibility allotted by these systems enables the user to adjust the assay based on their time, resources, and setting restrictions. Both fully integrated and customizable systems present a compelling and promising path for future development.

## Figures and Tables

**Table 1:** The number of journal articles (2020–present) demonstrated paper-based extraction, amplification + detection, detection, and fully integrated systems for various isothermal amplification tests. Color denotes the number of articles, with green being low and red being high.

	Extraction	Amplification + Detection	Detection	Fully Integrated	*Total*
RPA	4	3	14	1	22
RAA	0	0	10	0	10
MIRA	1	0	8	0	9
LAMP	0	6	6	5	17
RCA	0	2	12	0	14
NASBA	0	0	5	0	5
HDA	0	0	3	0	3
SDA	0	1	4	0	5
EXPAR	0	1	4	1	6
CAMP	1	0	0	0	1
*Total*	6	13	66	7	92

**Table 2:** The number of journal articles (2020–present) demonstrated paper-based detection (including amplification + detection and fully integrated systems) for numerous isothermal amplification tests, categorized by the detection methods. Color denotes the number of articles, with green being low and red being high. Additionally, all assays utilizing LFIA detection are indicated in parenthesis. Some articles demonstrated two or more detection methods and/or isothermal amplification tests in a single article, and they are double-counted. A more in-depth review of various assays is summarized in the Supplementary Data S1 (Excel sheet).

	Colorimetric	Fluorometric	Flow Distance	SERS	Other	*Total*
RPA	14 (14)	2	0	1	1	18 (14)
RAA	9 (9)	0	1	0	0	10 (9)
MIRA	8 (8)	0	0	0	0	8 (8)
LAMP	7 (5)	11	2	0	0	20 (5)
RCA	8 (7)	5 (1)	1	0	0	14 (8)
NASBA	5 (2)	0	0	0	0	5 (2)
HDA	3 (3)	0	0	0	0	3 (3)
SDA	3 (3)	1	1	0	0	5 (3)
EXPAR	3 (3)	2	0	0	1	6 (3)
CAMP	0	0	0	0	0	0
*Total*	60 (54)	21 (1)	5	1	2	89 (55)

## Data Availability

Not applicable.

## Supplementary Materials

The following supporting information can be downloaded at https://www.mdpi.com/article/10.3390/bios13090885/s1, Data S1: In-depth review of various assays (Excel sheet).
